# Frequency of Inappropriate Medication Prescription in Hospitalized Elderly Patients in Italy

**DOI:** 10.1371/journal.pone.0082359

**Published:** 2013-12-12

**Authors:** Francesco Napolitano, Maria Teresa Izzo, Gabriella Di Giuseppe, Italo F. Angelillo, the Collaborative Working Group, Collaborative Working Group, Vincenzo Castaldo, Andreo A De Stefano, Raffaele Dell'Aversano, Maurizio di Mauro, Vincenzo Iodice, Carmine Iovine, Giuseppe Matarazzo, Giacomo Rota, Antonio Sciambra

**Affiliations:** Hospital S. Giuseppe Moscati, Avellino; Hospital Santa Maria della Pietà, Nola, Naples; Hospital Pellegrini, Naples; Hospital San Paolo, Naples; Hospital of Marcianise, Caserta; Hospital SS. Anna e Sebastiano, Caserta; Hospital San Giovanni Bosco, Naples; Hospital S. Rocco, Sessa Aurunca, Caserta; Hospital Evangelico Villa Betania, Naples; Department of Experimental Medicine, Second University of Naples, Naples, Italy; University of British Columbia, Canada

## Abstract

**Background:**

Older people often need comprehensive treatment, including many medications, and polypharmacy is common. The aims of this cross-sectional investigation were to examine the potentially inappropriate medication during the hospitalization and to identify the factors that may influence such inappropriateness among elderly in Italy.

**Methods:**

A sample of 605 individuals aged 65 years and older admitted in non-academic public acute care hospitals was randomly selected. Prescription of inappropriate medications were evaluated during the period from the day of admission to a randomly preselected day (index day). Beers Criteria were used to evaluate appropriateness.

**Results:**

At least one potentially inappropriate medication prescription from the day of hospital admission to the index day has been observed in 188 patients (31.1%), and respectively 84.1% and 15.9% of them had received one or two inappropriate medications. A total of 15 medications was prescribed inappropriately to these 188 patients, for 215 times with a total of 1143 doses. The multivariate logistic regression analysis revealed that the significant predictors for having at least one potentially inappropriate medication prescription during the hospitalization were: patients having an elementary education level, a lower pre-admission performance-based measure of basic activities of daily living, having received an inappropriate drug before the hospitalization, a hospital stay in the general and in the specialties surgical wards, a longer length of hospital stay from the admission to the index day, and having received a higher number of drugs from the day of the hospital admission to the index day. The most prevalent inappropriate medications administered were ketorolac (27.4%), amiodarone (19.1%), and clonidine (11.2%).

**Conclusions:**

This study supports the need for clinical guidelines implementation to assist physicians in choosing the most appropriate drugs for the elderly and for effective education of all physicians.

## Introduction

It is well-established that in developed and developing countries the elderly account for a substantial portion of all health care resource use and medication costs in the light of the fact that multiple chronic and degenerative disorders are highly prevalent and are the major causes of disability and death [1], [2]. Elderly who have complex health problems often need more comprehensive treatment with a larger consumption of medications than any other age group and polypharmacy is commonly prescribed [3], [4]. Patients taking a large number of medications are more likely to have potentially inappropriate prescriptions, contributing to render the patients more vulnerable to undesirable drug-related problems, including drug-disease interactions, adverse effects, hospital admissions, and health resources utilization [5]–[11].

Inappropriate management of medicines is one explicit quality indicator related to drug utilization and is of particular concern in vulnerable populations such as the elderly. Experiences from various countries and healthcare systems in recent years have attempted to study the appropriate medicines prescription among elderly in different settings mainly ambulatory primary care [6], [12]–[16], nursing homes [17]–[20], community [21]–[26], and hospital [27]–[30]. To the authors' knowledge, however, there are little prevalence data in the literature regarding the prescription of inappropriate medicines among hospitalized elderly patients [31]–[35]. Therefore, the overall goals of this cross-sectional investigation were to examine the potentially inappropriate medications prescription during the hospitalization and to identify the factors that may influence such inappropriateness among elderly in Italy.

## Materials and Methods

During January 2011 through March 2012, a two stage cross-sectional study was carried out in a random sample of 18 medical and 16 surgical wards from 9 randomly selected non-academic public acute care hospitals in the area of Avellino, Caserta, and Naples (Italy). In this area, there are 44 hospitals, including respectively 426 and 281 medical and surgical wards. Before the study, all hospital's directors were contacted through invitation letter to present the study's protocol and to obtain their approval to conduct the survey. Data were collected on randomly preselected days and the recruitment days were rotated to ensure that all days of the week were sampled. All patients in the wards in the preselected days were screened by two well-trained physicians not involved in patient care to identify potential study subjects. To be eligible the patients had to meet the following inclusion criteria: (1) be aged 65 years or above; (2) stay on the ward for at least 24 hours; and (3) have received at least one medication from the day of admission. Participants who were unable to give informed consent for substantial cognitive impairment or were non-Italian speaking were excluded and there were no restrictions based on diagnoses. A total of 605 patients who satisfied the eligibility requirements were included consecutively and no eligible patients were excluded. The two physicians explained to each patient the purposes of the study, and a written informed consent was obtained before conducting the survey.

The sample size was determined before study initiation. It was estimated that approximately 20% of the patients would receive inappropriate medication prescription, a margin of error of 5%, a 95% confidence level, and a design effect of two was incorporated in the formula to cater for the design effect due to the cluster sampling method. Under the assumption of a 90% response rate, the total sample size was estimated at 547 participants.

The two physicians collected the data by a short face-to-face interview with each patient and by reviewing the medical record. The interview was developed in order to assess: socio-demographic characteristics, comprising age, gender, weight, height, educational attainment, and occupation; self-rated health status assessed on a ten-point Likert-type scale with responses ranging from 1 (poor) to 10 (excellent); number of hospital admissions in the preceding year; and pre-admission performance-based measure of independence in activities of daily living using the Katz Index [36]. The data collected from the medical record were the following: day of the hospital admission, ward of hospital stay, specific diagnoses, comorbidities quantified by the Charlson Comorbidity Index score [37], prescribed drugs (name, dosage, duration of the therapy) prior to hospital admission and from the day of admission to the index day, and length of hospital stay from the day of admission to the index day.

The frequency of inappropriate prescriptions was evaluated during the period from the day of admission to the index day. To define the use of medication as potentially inappropriate for older adults irrespective of medical conditions, the two physicians measured all medications prescribed in the above mentioned period by using the Beers updated explicit Criteria [38]. Fifty-three medications or medication classes are divided into three categories: potentially inappropriate medications and classes to avoid in older adults, potentially inappropriate medications and classes to avoid in older adults with certain diseases and syndromes that the drugs listed can exacerbate, and medications to be used with caution in older adults. The Beers updated Criteria have been used only for the drugs available in Italy.

The local ethical review board of the Second University of Naples, Naples (Italy) approved the protocol of the study according to the principles of the Declaration of Helsinki. All patients had received detailed information on the study, were informed that the participation was voluntary, and that all information gathered would be anonymous and handled confidentially. Participants had provided their signed informed consent prior to their inclusion.

### Statistical analysis

Following a descriptive analysis (frequencies) of the sample, univariate analysis was carried out using, where appropriate, a chi-squared test for categorical variables or an independent *t*-test for continuous variables in order to determine whether an association existed between the different characteristics and receiving a potentially inappropriate medication. Moreover, to account for the two-stage cluster sampling, a logistic regression model was estimated by using a Generalized Estimation Equation (GEE) analysis to account for clustering of the patients within hospital wards to assess the unique contribution of the variables to the outcome of interest, while controlling for all other variables included in the model. All the following variables regarding the patient were entered simultaneously in the model: age (continuous, in years), gender (male = 0; female = 1), educational level (four categories: no formal education = 0; elementary school = 1; middle school = 2; high school, baccalaureate/graduate degree = 3), ward of hospital stay in the index day (four categories: General Medicine = 1; Medical Specialties = 2; General Surgery = 3; Surgical Specialties = 4), length of hospital stay from the day of hospital admission to the index day (continuous, in days), pre-admission performance-based measure of Independence in Activities of Daily Living using the Katz Index (continuous), Charlson Comorbidity Index score (0–3 = 0; ≥4 = 1), having a hospital admission in the preceding year (no = 0; yes = 1), having received an inappropriate drug before the hospitalization (no = 0; yes = 1), continuation during the hospitalization of a prescription provided pre-hospitalization (no = 0; yes = 1), and number of drugs prescribed from the day of the hospital admission to the index day (<5 = 0; ≥5 = 1). Backward stepwise procedure was applied so that the final logistic model included only the characteristics providing a significant explanation of the outcome, in which the criterion for entering and for the elimination were the defaults of a *p*-value respectively of 0.2 and 0.4. The results of the regression analysis are expressed as odds ratios (ORs) and their 95% confidence intervals (CIs).

All *p*-values presented are two tailed and a criterion probability level of 0.05 or less was used to determine the statistical significance of associations between predictor variables and outcome. Statistical analyses were performed using Stata version 10.1 software [39].

## Results

All 605 patients who were eligible agreed to participate and were enrolled in the study giving an overall response rate of 100%. Table 1 described the main characteristics of the total sample and of those receiving a potentially inappropriate medication during the hospital stay and the findings for statistical associations. Participants were 51.9% males, aged between 65 and 103 years with an average of 76.7 years, only 13.4% had a high school or university education, 31.9% had a Charlson Comorbidity Index score greater of equal of four, the average of the Kats Index of Independence in Activities of Daily Living was 3.2 on a scale from 0 to 6 with a lower score indicating less success in independent living. In total, 39% had at least one hospital admission in the preceding year, 55.7% were hospitalized in medical wards, and to the majority were prescribed at least five drugs with a mean of 8.2. Only 13.2% of the patients had used at home at least once an inappropriate medication.

**Table 1:** Characteristics of the study population associated with the prescription of potentially inappropriate medications during the hospital stay.

		Total (*n* = 605)		Patients receiving at least one potentially inappropriate medication (*n* = 188)		
		n	%	n	%	
Gender	Male	314	51.9	94	50	
	Female	291	48.1	94	50	
Age (years)	65–69	100	16.5	28	14.9	
	70–74	163	26.9	47	25	
	75–79	133	22	44	23.4	
	80–84	111	18.4	42	22.3	
	≥85	98	16.2	27	14.4	
Education level	No formal education	103	17	27	14.4	
	Elementary school	326	53.9	111	59	
	Middle school	95	15.7	26	13.8	
	High school, Baccalaureate/Graduate degree	81	13.4	24	12.8	
Katz Index of Independence in Activities of Daily Livinĝ°*		3.2±2.7		2.8±2.6		
Charlson Comorbidity Index score°	0–3	412	68.1	124		65.9
	≥4	193	31.9	64		34.1
Number of hospital admissions in the preceding year	0	369	61	109		57.9
	≥1	236	39	79		42.1
Ward of hospital stay	General medicine	168	27.8	44		23.4
	Medical specialties	169	27.9	52		27.6
	General surgery	156	25.8	55		29.3
	Surgical specialties	112	18.5	37		19.7
Having received an inappropriate drug before the hospitalization*	No	525	86.8	132		70.2
	Yes	80	13.2	56		29.8
Continuation during the hospitalization of a prescription provided pre-hospitalization	No	432	71.4	129		68.6
	Yes	173	28.6	59		31.4
Number of drugs prescribed during hospital stay*	<5	96	15.9	15		7.9
	≥5	509	84.1	173		92.1
Length of hospital stay from the admission to the index day (median days)		6		7		

? Range from 0 to 6 with highest scores indicate complete independence.

° Range from 0 to 11 with highest scores indicate greater comorbidity.

° Mean±standard deviation.

* Statistically significant finding: *p*<0.05.

Among the 605 patients, 188 (31.1%) received at least one potentially inappropriate medication prescription according to the Beers Criteria from the day of hospital admission to the index day and, respectively, 84.1% and 15.9% of them had received one and two inappropriate medications. As shown in the univariate analyses, a significantly higher frequency of patients who have received at least one potentially inappropriate prescribed medication during the hospitalization has been observed among those with a lower pre-admission performance-based measure of basic activities of daily living using the Katz score (t = 2.56; 603 df; *p* = 0.01), those who have received an inappropriate drug before the hospitalization (χ^2^ = 65.22; 1 df; *p*<0.0001), those with a longer length of hospital stay (t = -4.18; 603 df; *p*<0.0001), and those with a higher number of drugs prescribed during the hospital stay (χ^2^ = 12.72; 1 df; *p*<0.0001). Generalized Estimation Equation analysis with a backward stepwise logistic regression model was performed to discern whether the different variables would predict potentially inappropriate medication prescribing and the results are reported in Table 2. The results of the multivariate regression were substantially similar to the bivariate associations and revealed that having an elementary education level (OR = 1.98; 95% CI 1.1–3.56), a lower pre-admission performance-based measure of basic activities of daily living (OR  =  0.89; 95% CI 0.82–0.97), having received an inappropriate drug before the hospitalization (OR = 10.34; 95% CI 5.75–18.59), a hospital stay in the general (OR = 1.87; 95% CI 1.09–3.22) and in the specialties surgical wards (OR = 1.83; 95% CI 1.01–3.3), a longer length of hospital stay from the day of admission to the index day (OR = 1.06; 95% CI 1.03–1.09), and having received a higher number of drugs during the hospitalization (OR = 3.45; 95% CI 1.78–6.69) were identified as independent and significant predictive factors for having at least one potentially inappropriate medication prescribed during the hospital stay.

**Table 2:** Potential predictors of potentially inappropriate medications prescribed during the hospital stay based on GEE model using multivariate logistic regression analysis.

Variable		OR	SE	95% CI	*p* value
Patients receiving at least one potentially inappropriate medication					
Number of drugs prescribed during the hospital stay		3.45	1.17	1.78–6.69	<0.001
Length of hospital stay from the admission to the index day (days)		1.06	0.02	1.03–1.09	<0.001
Having received an inappropriate drug before the hospitalization		10.34	3.09	5.75–18.59	<0.001
Katz Index of Independence in Activities of Daily Living		0.89	0.04	0.82–0.97	0.008
Highest educational level	No formal education	1.0*			
	Elementary school	1.98	0.59	1.1–3.56	0.023
	Middle school	1.46	0.57	0.68–3.14	0.33
	High school, Baccalaureate/Graduate degree	1.93	0.78	0.88–4.24	0.1
Ward of hospital stay	General medicine	1.0*			
	General surgery	1.87	0.52	1.09–3.22	0.023
	Surgical specialties	1.83	0.55	1.01–3.3	0.046
	Medical specialties	1.2	0.33	0.7–2.05	0.51
Age (years)		0.98	0.02	0.95–1.01	0.2
Continuation during the hospitalization of a prescription provided pre-hospitalization		0.82	0.19	0.52–1.29	0.39
Gender	Male	1.0*			
	Female	1.18	0.25	0.78–1.78	0.43

* Reference category.

The potentially inappropriate medications prescribed during the hospital stay are reported in Table 3. The applications of Beers updated explicit Criteria identified that a total of 15 medications were potentially inappropriately prescribed to the 188 patients, for 215 times with a total of 1143 doses. The most commonly medications were, in a decreasing order, analgesics (27.4%), antiarrhythmics (21.9%), and antihypertensive (20.9%). Ketorolac was the only analgesic drug inappropriately prescribed, whereas among the antiarrhythmics, amiodarone, digoxin, and propafenone were inappropriately prescribed respectively in 19.1%, 2.3% and 0.5% of the patients. Clonidine (11.2%) and doxazosin (7.4%) were the most prevalent inappropriate antihypertensive drugs.

**Table 3:** Potentially inappropriate medications prescribed during the hospital stay in the study population.

		Number of patients receiving potentially inappropriate medications (*n* = 188)*		Number of prescriptions of potentially inappropriate medications (*n* = 215)		Number of doses potentially inappropriate (*n* = 1143)	
		n	%	n	%	n	%
Analgesic/Anti-inflammatory	Ketorolac	59	31.4	59	27.4	120	10.5
Antiarrhythmics	Amiodarone	41	21.8	41	19.1	285	24.9
	Digoxin	5	2.6	5	2.3	41	3.6
	Propafenone	1	0.5	1	0.4	1	0.1
Antihypertensive	Clonidine	24	12.8	24	11.2	190	16.6
	Doxazosin	16	8.5	16	7.4	105	9.3
	Nifedipine	5	2.6	5	2.3	8	0.8
Anticlotting	Aspirin	22	11.7	22	10.2	117	10.3
	Ticlopidine	18	9.6	18	8.4	139	12.3
Antipsychotics	Promazine	8	4.2	8	3.7	28	2.4
	Haloperidol	3	1.6	3	1.4	16	1.4
Benzodiazepines	Diazepam	7	3.7	7	3.3	48	4.1
	Lorazepam	3	1.6	3	1.4	15	1.3
Laxative	Vaseline oil	2	1	2	0.9	13	1.1
Anticholinergic	Chlorpheniramine	1	0.5	1	0.4	15	1.3

* A total of 27 patients received two potentially inappropriate medications.

## Discussion

The present survey - the first of its kind in Italy - focuses on a growing public health problem the inappropriate medications prescription during the hospitalization in elderly and on the identification of the factors that may influence such inappropriateness.

A disturbingly high level of potentially inappropriate prescribed medications has been observed (31.1%) which is of concern, indicating room for improvement. Comparison with the results from previously studies conducted worldwide is not straightforward due to the variations in methods issues such as data and duration of collection, study criteria, and characteristics of the population groups. Despite these variations, this prevalence was considerably lower than those observed in the United States with values of 49% among elderly admitted with 1 or more of 7 common medical diagnoses [32] and of 55.3% in those undergoing surgery during their hospitalization [33] and of 56.1% in acute care hospitals in Japan [34]. In the current study, it has been found a higher rate of inappropriate prescriptions than have been reported in a tertiary care teaching hospital in India with a frequency of 24.6% [35], in wards of the Department of Medicine in Croatia with 22.1% [31], and among older emergency department patients in the United States with 16.8% [40] and in Taiwan with 14.7% [41]. Explanations of the differences probably may also be related to the organization of the health care delivery systems and to the availability of social support services.

The results described of the multivariate analysis about factors associated with a potentially inappropriate prescription showed that it is determined by many influential predictors, including the patient's socioeconomic level, the pre-admission basic activities of daily living, having received a drug before the hospitalization, the ward of the hospital stay, the length of hospital stay, and the number of drugs prescribed during their hospitalization. Patients who had a significantly higher rate of inappropriate prescription were those with a lower educational level, with a lower pre-admission performance-based measure of basic activities of daily living, who have received an inappropriate drug before the hospitalization, those in surgical wards, those with a longer length of hospital stay from the day of admission to the index day, and those who have received a higher number of drugs during the hospitalization. Some of these findings were in line with those from previous research. Indeed, in the already mentioned study conducted in Japan, although in the univariate analysis, the inappropriate drugs were prescribed more frequently in patients with a higher median hospitalized days and in surgical wards [34]. Moreover, in India the results of a logistic regression model showed that multiple diseases and a higher use of drugs during the hospital stay predicted potentially inappropriateness [35]. The significantly higher rate of inappropriate prescription among those who have received at least one inappropriate drug prior to hospital admission is in accordance with the observation amongst hospitalized elderly in South Australia [30]. A possible reason is that physicians at the admission and during the hospitalization rarely review the drugs that patients were taking pre-admission and, therefore, they do not modify the pharmacological treatment until they do not have certainty about the diagnosis. This suggests that a closer collaborative care between primary healthcare sector, specifically the general practitioners, and hospital clinicians is needed to provide higher quality of care. They must work together to build mutual rapport also about prescribed medicines and this may reduce the rate of inappropriate prescription. Also in the multivariate analysis, the number of drugs prescribed seems to be one of the most important independent predictors of receiving an inappropriate medication. Therefore, reducing the number of drugs administered to a patient is a possible way to diminish the prevalence of such inappropriateness. Finally, a low education level was significantly associated with an inappropriate medication prescription and this may be partially explained with the consideration that a low level has been found to negatively influence an individual's perception about their health status, knowledge about health related issues, and health-related behaviors, which could lead such individuals to be less prone to interact with physicians regarding the medical treatment. Indeed, the relationship between higher levels of socio-economic status and higher knowledge about health related issues has been well established [42]–[48].

As regards the most frequently prescribed medications identified as inappropriate, the present study revealed that the largest proportion belongs to the analgesic (27.4%), antiarrhytmics (21.9%), and antihypertensives (20.9%), and the most prevalent drugs were ketorolac (27.4%), amiodarone (19.1%), and clonidine (11.2%). These findings were consistent with previous studies [31], [32], [40], [49]. Although the evaluation of the adverse health outcomes was beyond the scope of this study, these inappropriate medications prescribed during the hospital stay should be of particular concern since they have a clinical impact due to the increased risk of adverse side effects. Indeed, ketorolac and aspirin have been observed to be associated with an increased risk of gastrointestinal adverse events [50]–[52], and amiodarone with cardiovascular, pulmonary, endocrine, and ophthalmologic adverse events [53]–[55]. These adverse outcomes underlined the need for a more careful attention in the prescriptions of drugs in this vulnerable population.

When interpreting the findings of the current study, it is worth mentioning some limitations to the methodology that could bias estimates. The first limitation lies with the fact that the study is cross-sectional, which does not allow us to determine the determinants associated with the complex phenomenon of the inappropriate medication prescription during the hospitalization are causal in nature. Second, the use of the Beers Criteria may have underestimated the frequency of inappropriateness since some medications, especially those not in use in the United States, are not included. Third, although the information was gathered mainly from the chart review, patients reported through a structured interview the medicines received before the admission, including the duration of treatment and the dosage, and recall bias may have been a problem. Fourth, this sample is unlikely to be representative of all of Italy because the study was conducted in one region of the country and, therefore, the ability to generalize the findings is questionable. However, the population of this area is similar to that of other Italian regions in terms of age, gender, and education. Even with these acknowledged study weaknesses, we believe that they do not undermine the results and conclusions.

In order to improve the medication prescription quality for the elderly, by reducing the frequency of using medications for which there is no clinical need during the hospitalization, clinical guidelines implementation, to assist physicians in order to deliver the medications that are appropriate, and education strategies are imperatively highlighted.
